# Dietary Interventions and Changes in Cardio-Metabolic Parameters in Metabolically Healthy Obese Subjects: A Systematic Review with Meta-Analysis

**DOI:** 10.3390/nu8080455

**Published:** 2016-07-28

**Authors:** Marta Stelmach-Mardas, Jarosław Walkowiak

**Affiliations:** 1 Department of Epidemiology, German Institute of Human Nutrition, Potsdam-Rehbruecke, Arthur-Scheunert-Allee 114-116, Nuthetal 14558, Germany; 2 Department of Pediatric Gastroenterology and Metabolic Diseases, Poznan University of Medical Sciences, Szpitalna Str 27/33, Poznan 60-572, Poland; jarwalk@ump.edu.pl

**Keywords:** metabolically healthy obese, diet, biomarkers

## Abstract

The aim of this systematic review was to assess the effect of diet on changes in parameters describing the body size phenotype of metabolically healthy obese subjects. The databases Medline, Scopus, Web of Knowledge and Embase were searched for clinical studies carried out between 1958 and June 2016 that reported the effect of dietary intervention on BMI, blood pressure, concentration of fasting triglyceride (TG), high density lipoprotein cholesterol (HDL-C), fasting glucose level, the homoeostatic model assessment of insulin resistance (HOMA-IR) and high sensitivity C-Reactive Protein (hsCRP) in metabolically healthy, obese subjects. Twelve clinical studies met inclusion criteria. The combined analyzed population consists of 1827 subjects aged 34.4 to 61.1 with a BMI > 30 kg/m^2^. Time of intervention ranged from eight to 104 weeks. The baseline characteristics related to lipid profile were more favorable for metabolically healthy obese than for metabolically unhealthy obese. The meta-analyses revealed a significant associations between restricted energy diet and BMI (95% confidence interval (CI): −0.88, −0.19), blood pressure (systolic blood pressure (SBP): −4.73 mmHg; 95% CI: −7.12, −2.33; and diastolic blood pressure (DBP): −2.75 mmHg; 95% CI: −4.30, −1.21) and TG (−0.11 mmol/l; 95% CI: −0.16, −0.06). Changes in fasting glucose, HOMA-IR and hsCRP did not show significant changes. Sufficient evidence was not found to support the use of specific diets in metabolically healthy obese subjects. This analysis suggests that the effect of caloric restriction exerts its effects through a reduction in BMI, blood pressure and triglycerides in metabolically healthy obese (MHO) patients.

## 1. Introduction

The prevalence of obesity is increasing worldwide with prognoses expected to affect more than one billion people by 2030 [1]. An association between obesity and increased risk of co-morbidities, i.e., metabolic syndrome (MetS) and cardiovascular disease (CVD) leading to significantly higher all-causes of mortality, has been observed [2]. However, the metabolically healthy obese (MHO) phenotype was described in the early 1980s [3], and, to date, there is no single consistent definition that covers “the metabolic health” approach in relation to differentiated dietary habits in obese subjects [4,5,6].

So far, the direct mechanism, which contributes to the different effects of weight loss in MHO and metabolically unhealthy obese (MUHO) subjects is not known, though it seems that it may vary as a function of different baseline metabolic profiles in MHO and MUHO groups. Only limited data are available with regards to dietary behaviors in MHO [7]. The prevalence of MHO is predicted to be 30%–40% of the obese population, with higher rates in younger subjects and in females [4,8]. Nevertheless, it should be taken under consideration that MHO subjects can shift to the metabolically unhealthy phenotype, a change confirmed by Schröder et al. [9] in a 10 years follow-up study. 

Due to the fact that the effectiveness of dietary interventions in MHO is not very well known, we aimed to describe the influence of applied diet from intervention studies on changes in parameters describing the body size phenotype (body mass index—BMI, blood pressure—BP and concentration of selected biomarkers) of MHO. 

## 2. Experimental Section

### 2.1. Search Strategy

The databases Medline, Scopus, Web of Knowledge and Embase were searched for clinical studies carried out between 1958 and June 2016 that reported the effect of dietary intervention on BMI and selected cardio-metabolic parameters (blood pressure and concentration of selected biomarkers) as primary or secondary outcomes in MHO. Search strategy was restricted to humans, English language and full length, original articles. The search based upon the listed below following index terms and title: #1 “Benign Obesity, Metabolically” OR “Metabolically Healthy Obesity” OR “Healthy Obesity, Metabolically” OR “Obesity, Metabolically Healthy” OR “Metabolically Benign Obesity” OR “Metabolically normal” OR “Metabolic syndrome” AND #2 ”Diet” OR “Diet, carbohydrate-restricted” OR “Diet, fat-restricted” OR “Diet, protein-restricted” OR “Ketogenic diet” OR “Diet, high-fat” OR “Diet, reducing” OR “Weight reduction programs” OR “Caloric restriction” OR “Lifestyle intervention” NOT “Animals”. The Preferred Reporting Items for Systematic Reviews and Meta-Analyses (PRISMA) Statement was followed [10]. 

### 2.2. Metabolically Healthy Obese—Definition

The criteria for MHO used in the studies include: absence of abdominal obesity on the basis of waist circumference, absence of metabolic syndrome components, e.g., normal blood pressure, normal lipid values, normal fasting glucose concentrations (at times also including normal C-reactive protein concentrations), insulin sensitivity determined on the basis of the homoeostatic model assessment of insulin resistance (HOMA-IR) and a high level of cardiorespiratory fitness [11]. All features were also used in the long-term prognosis of cardiovascular disease and all-cause mortality for MHO by Bo et al. [12], Calori et al., [13] and Hammer et al. [14].

Specifically, the criteria for the body size phenotype of MHO subjects include: a BMI ≥ 30 kg/m^2^ and <2 cardio-metabolic abnormalities (systolic/diastolic blood pressure (SBP/DBP) ≥ 130/85 mmHg or antihypertensive medication use; fasting triglyceride (TG) level ≥ 150 mg/dL (1.693 mmol/L)); decreased high density lipoprotein cholesterol (HDL-C) level < 40 mg/dL (1.0344 mmol/L) in men and <50 mg/dL (1.293 mmol/L) in women or lipid-lowering medication use; fasting glucose (Glc) level ≥ 100 mg/dL (5.55 mmol/L) or antidiabetic medication use; insulin resistance: HOMA-IR > 5.13, i.e., the 90th percentile; systemic inflammation: high sensitivity C-Reactive Protein (hsCRP) level > 0.1 mg/L, i.e., the 90th percentile) [6]. Similar criteria were proposed by Meigs et al. [5] including the addition of waist circumference (WC) (>102 cm in men and >88 cm in women) and expanding the definition to include up to <3 cardio-metabolic abnormalities. 

### 2.3. Inclusion and Exclusion Criteria

Only studies conducted with subjects described as metabolically healthy obese subjects, indicating the changes in BMI, blood pressure and selected blood parameters after various dietary interventions, were included. The intervention studies (randomized controlled trial and non-randomized trial) were taken into consideration. The articles that did not meet inclusion criteria (animal studies, other than the type of documents mentioned above, articles in any other language than English) were excluded. 

### 2.4. Data Extraction and Analysis

**Figure 1:**
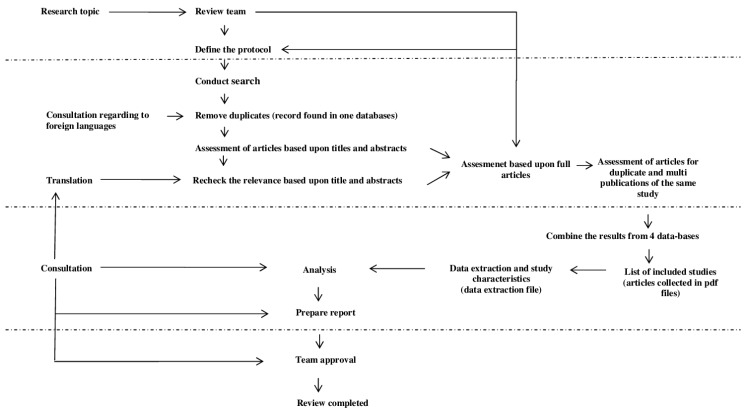
Process flow sheet.

Relevant articles were identified by screening the abstracts, titles and full-texts. The study selection process was performed by two independent researchers (M.S.-M. and J.W.) in parallel for each database. In every step of assessment, all disagreements between researchers were resolved after consultation. In the case of disagreement during the title assessment process, the paper was included in the next step. The process outline and workflow is presented in Figure 1.

Eligible studies were evaluated according to: the number of participants, study design, type of dietary intervention, changes in BMI (defined as body mass divided by the square of the height), and criteria for body size phenotype of MHO defined according to Third Report of the National Cholesterol Education Program Adult Treatment Panel (NCEP ATP III) [6]. To assess the study quality, a nine-point scoring system according to the Newcastle-Ottawa Scale was used. The maximum score was nine, with a high-quality study defined by a threshold of ≥7 points [15]. 

The recorded biomarkers concentrations were converted to mmol/L (fasting glucose, Triglycerides, HDL-Cholesterol) and mg/L (hsCRP) in order to standardize results. A meta-analysis was performed to combine the results of individual studies. Data were analyzed using a random-effects model. The effect size of a study was investigated by calculating the standardized mean difference with a 95% confidence interval (CI). The heterogeneity of the sum of studies was tested for significance. As a measure for quantifying inconsistency, *I*^2^ was selected [16]. Although included studies in our analysis were heterogeneous, careful inclusion of the suited arms (MHO group) in different interventions allowed us to combine the collected papers and run our analyses. The results of the meta-analysis were visualized using a forest plot which illustrates the results of the individual studies and the summary effect. The analysis was performed with Review Manager (RevMan, V5.3, The Nordic Cochrane Centre, the Cochrane Collaboration, Copenhagen, Denmark, 2014).

## 3. Results

### 3.1. Search Results

**Figure 2:**
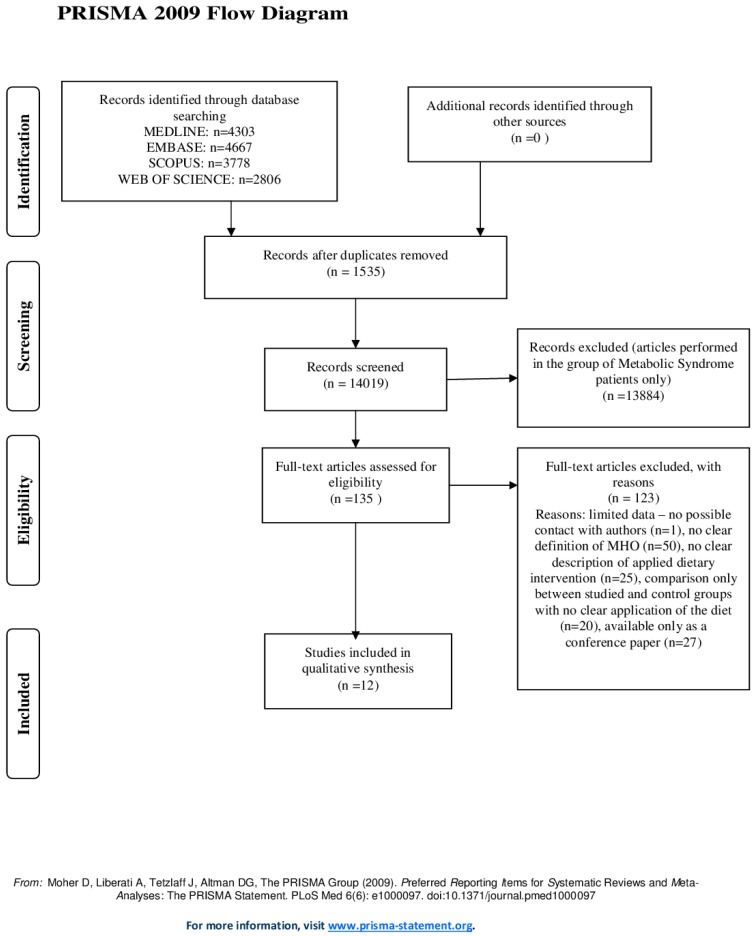
Process of literature search on the association between diet and selected cardio-metabolic parameters in metabolically healthy obese.

Using wide terms to describe the metabolically healthy obese patients, we end up with more than 14,000 articles that were screened. After initial exclusion criteria, 135 papers were assigned for full-text review with 12 articles included for data extraction and analysis [17,18,19,20,21,22,23,24,25,26,27,28] (Figure 2). 

### 3.2. Studies and Populations Characteristics

The characteristics of clinical studies (randomized and non-randomized) and populations are presented in Table 1. The population consists of 1827 subjects and was characterized by a baseline BMI > 30 kg/m^2^, a mean age from 34.4 [18] to 61.1 [20], and representing Caucasian and Asian ethnicities. Time of interventions ranged from 12 to 104 weeks and were based on diet only [18,21,22,23,24,25,26,27] or diet with combination of light to moderate physical activity (PA) [19,20,28] that supported the daily energy deficit. 

### 3.3. Changes in Body Mass Index and Selected Cardio-Metabolic Outcomes during Dietary Interventions

**Figure 3:**
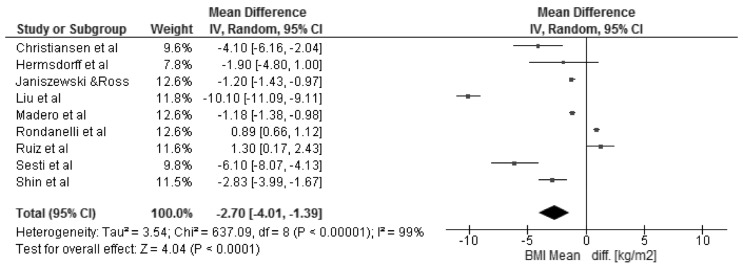
Forest plot of the random-effects meta-analysis of changes in BMI according to reduction in energy intake shown as polled standard differences in the means with 95% Cis and in randomized and non-randomized trials. * For each study, the square represents the point estimate of the intervention effect. Horizontal lines join the lower and upper limits of the 95% CI of this effect. The area of shaded squares reflects the relative weight of the study in the meta-analysis. Diamonds represent the subgroup mean difference and pooled mean differences. CI indicates confidence interval.

**Figure 4:**
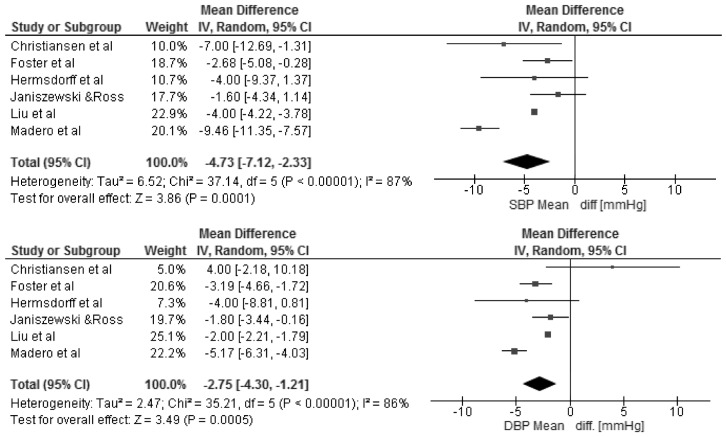
Forest plot of the random-effects meta-analysis of changes in Systolic and Diastolic Blood Pressure according to reduction in energy intake shown as polled standard differences in the means with 95% Cis and in randomized and non-randomized trials. * For each study, the square represents the point estimate of the intervention effect. Horizontal lines join the lower and upper limits of the 95% CI of this effect. The area of shaded squares reflects the relative weight of the study in the meta-analysis. Diamonds represent the subgroup mean difference and pooled mean differences. CI indicates confidence interval.

**Figure 5:**
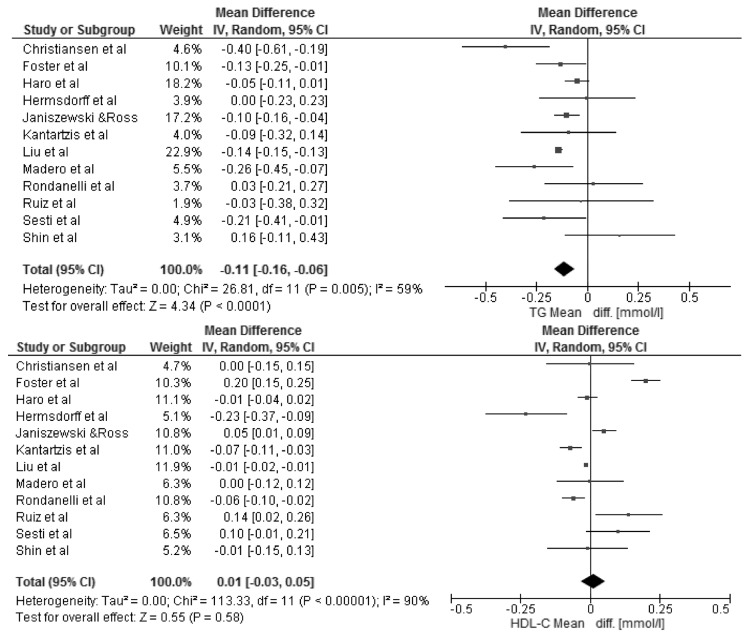
Forest plot of the random-effects meta-analysis of changes in Triglycerides and HDL-cholesterol according to reduction in energy intake shown as polled standard differences in the means with 95% Cis and in randomized and non-randomized trials. * For each study, the square represents the point estimate of the intervention effect. Horizontal lines join the lower and upper limits of the 95% CI of this effect. The area of shaded squares reflects the relative weight of the study in the meta-analysis. Diamonds represent the subgroup mean difference and pooled mean differences. CI indicates confidence interval.

**Figure 6:**
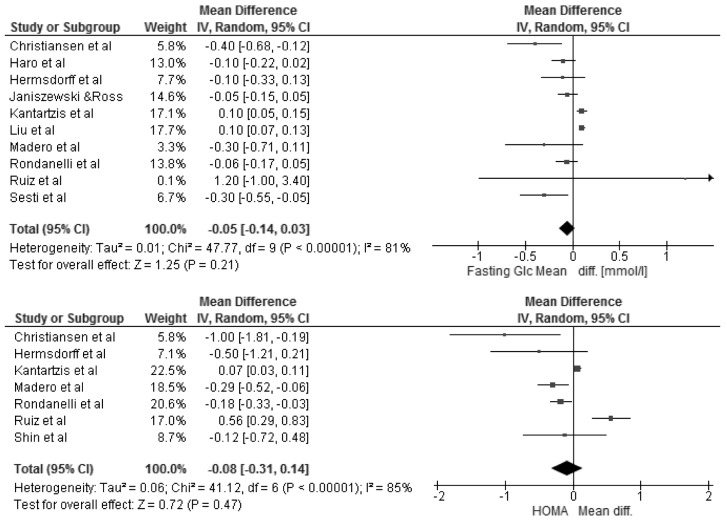
Forest plot of the random-effects meta-analysis of changes in Fasting Glucose and HOMA-IR according to reduction in energy intake shown as polled standard differences in the means with 95% Cis and in randomized and non-randomized trials. * For each study, the square represents the point estimate of the intervention effect. Horizontal lines join the lower and upper limits of the 95% CI of this effect. The area of shaded squares reflects the relative weight of the study in the meta-analysis. Diamonds represent the subgroup mean difference and pooled mean differences. CI indicates confidence interval.

**Figure 7:**
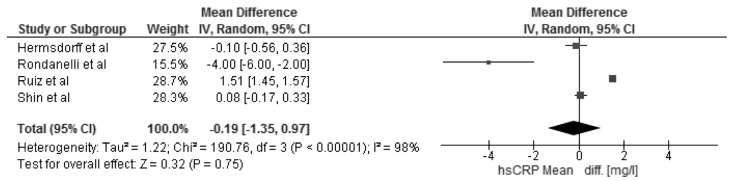
Forest plot of the random-effects meta-analysis of changes in high sensitivity C-Reactive Protein according to reduction in energy intake shown as polled standard differences in the means with 95% Cis and in randomized and non-randomized trials. * For each study, the square represents the point estimate of the intervention effect. Horizontal lines join the lower and upper limits of the 95% CI of this effect. The area of shaded squares reflects the relative weight of the study in the meta-analysis. Diamonds represent the subgroup mean difference and pooled mean differences. CI indicates confidence interval.

The changes in BMI and selected cardio-metabolic outcomes during dietary interventions are presented in Table 2. Reduction in BMI, from baseline to the final day of intervention, ranged from 1.1 to 2.9 kg/m^2^ in MHO, and were statistically significant in seven of twelve studies [18,20,22,23,25,27,28] within the study group. Kantartizis et al. [19], Haro et al. [24], and Foster et al. [26] failed to report exact values of baseline BMIs. The quantitative meta-analysis revealed a significant association between the restricted energy diets (*p* < 0.0001, *I*^2^ = 99%) and change in BMI (−2.70 kg/m^2^; 95% CI: −4.01, −1.39) (Figure 3). The changes in systolic and diastolic blood pressure during the dietary interventions were measured only in six studies [20,22,25,26,27,28]. However, the meta-analysis showed statistically significant reduction in SBP (−4.73 mmHg; 95% CI: −7.12, −2.33; *p* = 0.0001, *I*^2^ = 87%) and DBP (−2.75 mmHg; 95% CI: −4.30, −1.21; *p* = 0.0005, *I*^2^ = 86%) within MHO group after applied dietary interventions, clinical relevance cannot be considered (Figure 4). The concentrations of TG and HDL-C were reported in all selected studies, where the baseline characteristic with regards to blood lipids was mostly more favorable for MHO than for metabolically unhealthy obese (MUHO) [19,21,23]. Nevertheless, the statistically significant association was observed only between energy restricted diets and the reduction in TG concentration (−0.11 mmol/L; 95% CI: −0.16, −0.06; *p* < 0.0001, *I*^2^ = 59%) (Figure 5). Fasting glucose was assessed in ten studies [15,16,17,18,20,21,22,23,25,26] with no significant decrease (−0.05 mmol/L; 95% CI: −0.14, 0.03; *p* = 0.21, *I*^2^ = 81%). Additionally, in the meta-analysis of studies reporting changes in HOMA-IR [17,18,19,21,25,27,28] in relation to dietary intervention, no significant reduction was observed within MHO group (−0.08; 95% CI: −0.31, 0.14; *p* = 0.47, *I*^2^ = 85%) (Figure 6). The reduction in hsCRP concentration were reported in only four studies [17,18,21,27] with no significant association with dietary intervention found (−0.19 mg/L; 95% CI: −1.35, 0.97; *p* = 0.75, *I*^2^ = 98%) (Figure 7). The funnel plot did not reveal asymmetry despite selected studies being outliers, suggesting no real evidence of a publication bias (Figure S1).

## 4. Discussion

Here, we present the first review summarizing the results from clinical studies performed in MHO with a primary interest in changes in BMI and selected cardio-metabolic outcomes. The findings of the conducted systematic review with meta-analysis did not find sufficient evidence to support the use of some specific diet in metabolically healthy obese subjects. However, it seems that effect of caloric restriction is related to reduction in BMI, blood pressure and triglycerides in the group of MHO patients. 

It was imperative for the conducted review to show that the MHO subjects should be recognized as the core group with a primary interest in a changing lifestyle being the determinant of “metabolic health”. Other longitudinal studies [29,30] with shorter follow-up (6–8.2 years) have indicated that only approximately half of MHO subjects maintained their “metabolic health” status. It was also suggested that initially MHO subjects undergo adverse metabolic changes associated with obesity over time [31]. Nevertheless, there is no clear data regarding the most beneficial dietary interventions, nor the effectiveness, of energy restricted diets in MHO patients taking into account clinical significance.

As previously demonstrated [32,33], obesity can be associated with a higher relative risk for CVD and cancer mortality compared to non-obese subjects. Therefore, the decrease in BMI can reflect the improvement in body composition, and consequently, reduce the mortality [34]. We have observed a significant association between the applications of energy restricted diets and BMI reduction in MHO individuals. Recently, Phillips and Perry [35] has also indicated that greater low density lipoprotein cholesterol (LDL-C) and HDL-C and less very low density lipoprotein cholesterol (VLDL-C) particles increase the likelihood of MHO. However, the results of our meta-analysis, through the applied dietary interventions, indicated only significantly beneficial association with the TG concentration within the MHO group. However, the inflammatory status in MHO can be reduced [36], partially stemming from more favorable fatty acid profiles [37] compared to MUHO, a significant decrease in hsCRP concentration was not observed in our analysis. As has been highlighted by Karelis et al. [38] health status may strongly influence the response to diet. For example, it was indicated in the MHO sedentary obese postmenopausal women without type 2 diabetes that the response to an energy-restricted diet may be different compared to at-risk individuals who achieve a similar weight loss, in that insulin sensitivity significantly improved in at-risk participants, but significantly deteriorated in MHO individuals in response to long-term diet. In our study, we did not find significant relation between energy restricted diets and biomarkers related to carbohydrates metabolism, i.e., fasting glucose and HOMA-IR within the MHO group of individuals. As shown in previously published studies [31,33], the baseline characteristic of metabolic profile can be more favorable in MHO compared to MUHO, which stay in line with some data selected for this systematic review [19,21]. As reported in Karelis et al. [39], MHO may have higher insulin sensitivity and a more favorable lipid profile. It has also been confirmed by Badoud et al. [40] that MHO individuals may show preserved insulin sensitivity and a greater ability to adapt to a caloric challenge compared to MUHO individuals. The postprandial response (i.e., area under the curve, AUC) for serum glucose and insulin were similar between MHO and lean healthy individuals and significantly lower than MUHO individuals (*p* < 0.05) [32]. However, a healthy metabolic profile and the absence of diabetic risk factors did not protect young adults from incident diabetes associated with overweight and obesity [40]. The intake of contraceptive pills by premenopausal women studied by Ruiz et al. [18] suggested that obtained results might be applied only for this sex and age group. Interestingly, the potential differences in body composition between MHO and MUHO were assessed in adults from the Pennington Center Longitudinal Study showing differences consistent between genders in both analyzed groups [34]. In clinical practice, as a goal for reducing CVD risk, the importance of body weight loss contributing to the maintenance of body weight loss is highlighted [4,41,42]. Camhi et al. [43] indicated that MHO young women demonstrate healthier lifestyle habits with less sedentary behavior, more time doing light physical activity, and healthier dietary quality for fat type and fiber in comparison to MUHO. Therefore, MUHO subjects are characterized by a higher risk of diabetes compared to MHO, which has been previously observed in Korean populations [44]. Indeed, metabolic health status, obesity and weight change were all independently associated with increased incidences of diabetes over five years of follow-up [44]. Nevertheless, we focused in our analysis on the MHO group of individuals only looking at the consequence of the applied dietary interventions. The data from the International Population Study on Macro/Micronutrients and Blood Pressure (INTERMAP) cohort study did not support the hypothesis that diet composition accounts for the absence of cardio-metabolic abnormalities in MHO [45]. Furthermore, it has also been confirmed by Kimokoti et al. [46] in the cross-sectional analysis from the REasons for Geographic And Racial Differences in Stroke (REGARDS) study. Although HOMA-IR index can be slightly higher in MHO subjects than in subjects characterized by normal weight, it may also be lower compared to MUHO [21]. It has been also shown that a long-term intensive lifestyle program, including Mediterranean diet nutritional counselling and high-intensity interval training, may be an appropriate intervention in MHO and MUHO subjects with similar potential clinical health benefits including an improved body composition, blood pressure, fasting glucose levels, insulin sensitivity, peak oxygen uptake, and muscle endurance [47]. Therefore, it could be beneficial that the results obtained in intervention studies with regards to analyzed cardio-metabolic outcomes may have yielded stronger and more consistent support from the results of observational studies. More broadened interventional studies are needed to assess different dietary approaches in MHO subjects.

## 5. Limitations

The present findings are based on limited ethnicity (Caucasian, Asian), and, therefore, results could vary as a function of ethnic background. Although the duration of interventions in analyzed studies was relatively long, we could not analyze long-term follow-up changes of analyzed cardio-metabolic parameters (no data available in the literature). Although the applied dietary interventions were very different and heterogeneous in nature, all of them were based on energy restriction. 

## 6. Conclusions

Based on the limited body of extracted data, we did not find sufficient evidence to support the use of some specific diet in metabolically healthy obese subjects. In general, it seems that the effect of caloric restriction is related to reduction in BMI, blood pressure and triglycerides in MHO individuals. 

## Figures and Tables

**Table 1:** Characteristics of the included studies and study populations.

Study	Country	Total Number (Number of MHO)	Age (Years) Mean ± SD	Study Design	Intervention	Time of Intervention	Study Quality (Newcastle-Ottawa Scale)
Rondanelli et al., 2015 [17]	Italy	MHO: 103	MHO: 42.2 ± 9.2	Clinical study: non-RCT	Low-energy mix, well-balanced (55% carbohydrates, 30% lipids and 15% proteins) diet providing 600 kcal less than individually estimated energy requirements based on the measured Resting Energy Expenditure	8-week	7
Ruiz et al., 2013 [18]	Spain	78 (MHO: 25)	MHO: 34.4 ± 6.8 MUHO: 37.8 ± 6.9	Clinical study: non-RCT	Low-energy mixed diet (55% carbohydrates, 30% lipids and 15% proteins) providing 600 kcal less than individually estimated energy requirements based on measured resting metabolic rate (RMR) and multiplied by a factor of 1.3, corresponding to a low physical activity level	12-week	8
Kantartzis et al., 2011 [19]	Germany	262 (MHO: 26)	MHO: 46.8 ± 2.2 MUHO: 47.1 ± 1.3	Clinical study: non-RCT	Lifestyle intervention program with aim to reduce body weight by ≥5%, to reduce the intake of energy from fat to <30% and particularly the intake of saturated fat to ≤10% of energy consumed and to increase the intake of dietary fiber to at least 15 g/4184 kJ (1000 kcal). Moderate sports per week: at least 3 h	36-week	7
Janiszewski & Ross, 2010 [20]	Canada	106 (MHO: 63)	MHO a. women: 61.1 ± 12.0 b. men: 61.4 ± 11.8 MUHO a. women: 46.5 ± 10.7 b. men: 53.1 ± 14.8	Clinical study: RCT	Men: program (diet or exercise) designed to induce a daily 700-kcal energy deficit Women: program (diet or exercise) designed to induce a daily 500-kcal energy deficit	Men: 12-week Women: 14-week	7
Shin et al., 2006 [21]	Korea	129 (MHO: 23)	MHO: 36.4 ± 11.2 MUHO: 39.8 ± 13.3	Clinical study: non-RCT	Weight loss program consisting of a 300 kcal/day reduction of usual caloric intakes to achieve the goal of losing a minimum of 3% of initial body weight	12-week	7
Liu et al., 2012 [22]	Canada	392 (MHO: 83)	53.6 ± 12.3	Clinical study: non-RCT	Calorie restricted meal plan of approximately 500–1000 calories below the patient’s baseline daily caloric requirement to achieve the goal of losing a 5% of initial body weight	>12-week	8
Sesti et al., 2011 [23]	Italy	190	MHO: 38 ± 10 MUHO: 40 ± 10	Clinical study: non-RCT	Diet applied after Laparoscopic Adjustable Gastric Banding First month: a semiliquid diet of 800 and 950 kcal/day in women and men, respectively (33% proteins, 19% lipids, 48% carbohydrates). Second month: a solid diet was reintroduced. Third month: the suggested diet was 970 and 1090 kcal/day in women and men, respectively (diet included 48% carbohydrates (starch or bread), 33% proteins (fat-free parts of different animals and fish), and 19% lipids (olive oil)	>12-week	7
Haro et al., 2015 [24]	Spain	MHO: 20	The LFHCC diet (a low-fat, high-complex carbohydrate diet) 61.4 ± 2.6 The Med diet (Mediterranean diet) 65.2 ± 3.2	Clinical study: RCT	The LFHCC diet contained 28% fat (12% monounsaturated; 8% polyunsaturated and 8% saturated) The Med diet contained 35% fat (22% monounsaturated; 6% polyunsaturated and 7% saturated)	52-week	8
Madero et al., 2011 [25]	Mexico	MHO: 131	The low-fructose diet 37.56 ± 1.14 The moderate natural fructose diet 40.15 ± 1.01	Clinical study: RCT	Energy-restricted diets: I. The low-fructose diet: first 2-week period of less than 10 g of fructose per day followed by a 4-week period of less than 20 g of fructose per day. II. The moderate natural fructose diet: consisted of 50 to 70 g of fructose consisting of mostly natural fructose from fruits.	6-week	7
Foster et al., 2010 [26]	US	MHO: 307	A low-carbohydrate diet 46.2 ± 9.2 A low-fat diet 44.9 ± 10.2	Clinical study: RCT	A low-carbohydrate diet which consisted of limited carbohydrate intake (20 g/day for 3 months) in the form of low-glycemic index vegetables with unrestricted consumption of fat and protein. After 3 months, participants in the low-carbohydrate diet group increased their carbohydrate intake (5 g per week) until a stable and desired weight was achieved A low-fat diet consisted of limited energy intake (1200 to 1800 kcal/day; ≤30% calories from fat)	104-week	8
Hermsdorff et al., 2011 [27]	Spain	MHO: 30	36.0 ± 8.0	Clinical study: RCT	The macronutrient-balanced diets (control and legume-based dietary approaches) were designed to provide a similar macronutrients distribution: 53% of energy as carbohydrates, 17% as proteins and 30% as fat	4-week	6
Christiansen et al., 2011 [28]	Denmark	MHO: 79	DIO group: 35.6 ± 7.0 DEX group: 37.5 ± 8.0	Clinical study: RCT	A liquid, very low energy diet of 600 and 800 kcal/day, respectively (proteins 41 g, carbohydrates 29 g, fat 5.6 g per 100 g), for 8 week followed by a weight maintenance diet for 4 week. In Diet-induced weight loss using a very low energy diet (DIO) and exercise and diet-induced weight-loss combined (DEX) groups the subjects should obtain similar weight losses to observe the possible specific, weight-independent effect of exercise. Thus, the subjects in the DEX group were allowed to consume 150–200 kcal more per day than the DIO group, reflecting the estimated extra energy expenditure of 1500 kcal/week during exercise activity. The supervised aerobic exercise three times per week with duration of 60–75 min per training session, with an estimated energy expenditure of 500–600 kcal per session	8-week	7

MHO: metabolically healthy obese; MUHO: metabolically unhealthy obese; non-RCT: non randomized control trial; LFHCC diet: a low-fat, high-complex carbohydrate diet; the Med diet: Mediterranean diet; DIO: a very low energy diet; DEX: exercise and diet-induced weight-loss combined.

**Table 2:** Changes in Body Mass Index and parameters describing cardio-metabolic outcomes in metabolically healthy obese.

Study	Intervention	Groups	BMI (kg/m^2^) Mean ± SD		Systolic/Diastolic Blood Pressure (mmHg) Mean ± SD		TG (mmol/L) Mean ± SD		HDL-C (mmol/L) Mean ± SD		Fasting Glucose (mmol/L) Mean ± SD		HOMA-IR Mean ± SD		hsCRP (mg/L) Mean ± SD	
			B ′	I ″	B ′	I ″	B ′	I ″	B ′	I ″	B ′	I ″	B ′	I ″	B ′	I ″
Rondanelli et al., 2015 [17]	Diet ONLY	MHO	0.89 (0.66 to 1.12)		-		0.03 (−0.21 to 0.27) *		−0.06 (−0.10 to 0.02) *		−0.06 (−0.17 to 0.05) *		−0.18 (−0.33 to 0.52) *		−4.00 (−6.00 to −1.00) *	
Ruiz et al., 2013 [18]	Diet ONLY	MHO:	+2.88 ± 1.3 *		-		–0.03 ± 0.9 *		+0.14 ± 0.3 *		+1.2 ± 5.6 *		+0.56 ± 0.7 *		+1.51 ± 0.15 *	
		MUHO:	+3.08 ± 1.1 *				+0.31 ± 0.79 *		+0.20 ± 0.28 *		+2.1 ± 6.4 *		+0.59 ± 0.66 *		+0.38 ± 1.88	
Kantartzis et al., 2011 [19]	Diet AND exercise	MHO:	>30		-		1.71 ± 0.41	1.62 ± 0.44	1.37 ± 0.08	1.30 ± 0.05	5.07 ± 0.08	5.17 ± 0.10	1.16 ± 0.06	1.23 ± 0.08	-	
		MUHO:					1.56 ± 0.12	1.49 ± 0.08	1.27 ± 0.03	1.22 ± 0.03	5.42 ± 0.06	5.26 ± 0.06	2.98 ± 0.13	2.44 ± 0.14		
Janiszewski & Ross, 2010 [20]	Diet OR exercise	MHO:											-		-	
		Men	−1.3 ± 1.0 *		−3.0 ± 11.0 */−2.1 ± 6.4 *		−0.2 ± 0.4 *		+0.1 ± 0.1 *		−0.1 ± 0.4 *					
		Women	−1.1 ± 0.8 *		−0.1 ± 11.3 */−1.5 ± 7.1 *		0.0 ± 0.3 *		0.0 ± 0.2 *		0.0 ± 0.4					
		MUHO:														
		Men	−1.9 ± 0.9 *		−2.1 ± 11.9 */−2.9 ± 10.4 *		−0.5 ± 0.7 *		+0.1 ± 0.1 *		−0.6 ± 0.7 *					
		Women	−1.8 ± 1.0 *		−1.9 ± 18.0 */0.3 ± 9.9 *		−0.3 ± 0.5 *		−0.0 ± 0.1 *		−0.3 ± 0.8 *					
Shin et al., 2006 [21]	Diet ONLY	MHO:	−2.83 ± 2 .74 **		-		1.09 ± 0.37	1.25 ± 0.54	1.33 ± 0.24	1.32 ± 0.25	-		1.80 ± 1.27	1.68 ± 0.76	0.74 ± 0.41	0.82 ± 0.45
		MUHO:	−3.16 ± 4.08 **				1.72 ± 0.73	1.54 ± 0.78	1.09 ± 0.26	1.16 ± 0.26			2.60 ± 1.61	2.40 ± 2.3	1.9 ± 1.98	1.50 ± 1.3
Liu et al., 2012 [22]	Diet AND Supporting education	MHO:														
		<5% BW loss	−0.2 ± 3.4		−8.0 ± 1.0		−0.03 ± 0.07		0.08 ± 0.03		0.1 ± 0.15					
		>5% BW loss	−10.1 ± 4.6		−4.0 ± 1.0		−0.14 ± 0.07		−0.015 ± 0.03		0.0 ±0.15					
		MUHO:														
		<5% BW loss	−1.1 ± 3.1		−4.0 ± 1.0		−0.19 ± 0.05		0.05 ± 0.01		−0.16 ± 0.09					
		>5% BW loss	−11.4 ± 5.6		−2.0 ± 1.0		−0.02 ± 0.05		0.02 ± 0.01		−0.07 ± 0.09					
Sesti et al., 2011 [23]	Diet Applied after LAGB ′′′	MHO:	41.1 ± 5.5	35.0 ± 5.3	-		1.34 ± 0.60	1.13 ± 0.52	1.24 ± 0.31	1.34 ± 0.31	5.2 ± 0.7	4.9 ± 0.7	-		-	
		MUHO:	44.0 ± 6.4	38.2 ± 5.6			1.58 ± 0.78	1.30 ± 0.55	1.27 ± 0.31	1.32 ± 0.34	5.7 ± 0.8	5.3 ± 0.7				
Haro et al., 2015 [24]	Diet ONLY	MHO:		-		-							-		-	
		LFHCC diet #	31.6 ± 0.8		129 ± 9.4		1.16 ± 0.09	1.11 ± 0.09	1.04 ± 0.06	1.03 ± 0.05	5.2 ± 0.2	5.1 ± 0.2				
		Med diet §	32.8 ± 0.5		136 ± 3.7		1.18 ± 0.13	0.97 ± 0.13	1.09 ± 0.06	1.16 ± 0.05	5.1 ± 0.2	5.4 ± 0.2				
Madero et al., 2011 [25]	Diet ONLY	MHO:													-	
		A low-fructose diet	−1.18 ± 0.82		−9.46 ± 7.77/−5.17 ± 4.69		−0.26 ± 0.78		0.0 ± 0.49		−0.30 ± 1.70		−0.29 ± 0.93			
		A moderate natural fructose diet	−1.57 ± 1.08		−7.85 ± 8.73/−6.04 ± 5.40		−0.35 ± 0.62		0.0 ± 0.31		−0.4 ± 0.5		−0.37 ± 0.57			
Foster et al., 2010 [26]	Diet AND Supporting education	MHO:	-								-		-		-	
		A low-carbohydrate diet			−2.68 (−5.08 to −0.27)/−3.19 (−4.66 to −1.73)		−0.13 (−0.25 to −0.01)		0.20 (0.15 to 0.25)							
		A low-fat diet			−2.59 (−5.07 to −0.12)/−0.50 (−2.13 to 1.13)		−0.16 (−0.28 to −0.03)		0.10 (0.08 to 0.16)							
Hermsdorff et al., 2011 [27]	Diet ONLY	MHO:														
		Calorie-restricted legume-free diet	31.3 ± 4.0	29.4 ± 4.1	115 ± 9/76 ± 9	111 ± 12/72 ± 10	1.17 ± 0.32	1.17 ± 0.57	1.50 ± 0.26	1.27 ± 0.31	5.1 ± 0.5	5.0 ± 0.4	2.1 ± 1.7	1.6 ± 1.0	2.0 ± 1.0	1.9 ± 0.8
		Calorie-restricted legume-based diet	33.7 ± 4.7	31.7 ± 3.9	115 ± 13/76 ± 6	106 ± 10/70 ± 6	1.11 ± 0.43	1.09 ± 0.42	1.27 ± 0.26	1.14 ± 0.18	5.2 ± 0.3	5.1 ± 0.3	1.8 ± 0.9	1.6 ± 0.9	2.7 ± 2.4	1.6 ± 0.9
Christiansen et al., 2011 [28]	Diet OR Exercise OR Diet with Exercise	Exercise only (EXO)	33.3 ± 4	32.2 ± 4	126 ± 15/76 ± 12	118 ± 8/68 ±9	1.6 ± 0.7	1.5 ± 0.4	1.3 ± 0.4	1.3 ± 0.5	5.6 ± 0.4	5.6 ± 5	2.3 ± 1.0	1.8 ± 1.0	-	
		Diet-induced weight loss using a very low energy diet (DIO)	35.3 ± 4	31.2 ± 4	129 ± 10/78 ± 12	122 ± 12/82 ± 12	1.5 ± 0.5	1.1 ± 0.3	1.2 ± 0.3	1.2 ± 0.3	5.5 ± 0.6	5.1 ± 0.5	3.1 ± 2.0	2.1 ± 1.0		
		Exercise and diet-induced weight-loss combined (DEX)	34.2 ± 3	30.3 ± 3	140 ± 17/82 ± 12	129 ± 18/72 ± 13	1.8 ± 0.6	1.2 ± 0.5	1.2 ± 0.3	1.3 ± 0.3	5.6 ± 0.4	5.4 ± 0.5	3.2 ± 2.0	2.0 ± 1.0		

′ B—Baseline; ′′ I—Intervention; * absolute changes; ** percent changes, statistically significant *p* < 0.05; MHO: metabolically healthy obese; MUHO: metabolically unhealthy obese; BMI: Body Mass Index; TG: triglycerides; HDL-C: high density lipoprotein cholesterol; HOMA-IR: homeostatic model assessment of insulin resistance; hsCRP: high-sensitivity C-reactive protein; BW—body weight changes; ′′′ LAGB: Laparoscopic Adjustable Gastric Banding; # The LFHCC diet (a low-fat, high-complex carbohydrate diet); § The Med diet (Mediterranean diet) 65.2 ± 3.2.

## Supplementary Materials

The following are available online at http://www.mdpi.com/2072-6643/8/8/455/s1, Figure S1: Funnel plot of standard error by standard differences in means of (a) Body Mass Index; (b) Systolic Blood Pressure; (c) Diastolic Blood Pressure; (d) Tryglicerides; (e) HDL-cholesterol; (f) Fasting Glucose; (g) HOMA-OR; (h) hsCRP.
