# Supplementary material for: Dietary Interventions and Changes in Cardio-Metabolic Parameters in Metabolically Healthy Obese Subjects: A Systematic Review with Meta-Analysis
Source: Nutrients. 2016 Jul 28;8(8):455. doi: 10.3390/nu8080455 (PMC4997370; doi:10.3390/nu8080455)
Supplement: Supplementary file 1 [file nutrients-08-00455-s001.doc]

**Supplematary Materials: Dietary Interventions and Changes in Cardio-Metabolic Parameters in Metabolically Healthy Obese Subjects: A Systematic Review with Meta-Analysis**

**Marta Stelmach-Mardas and Jarosław Walkowiak**

| 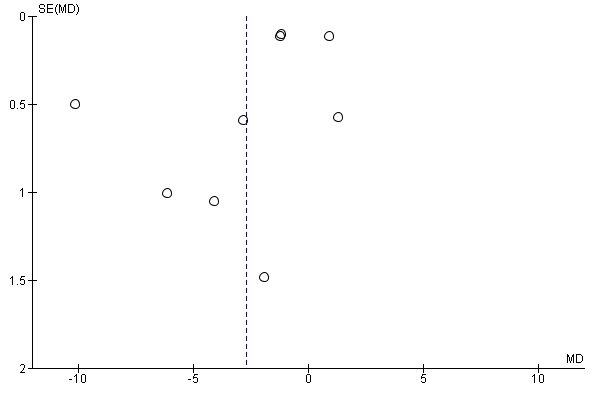 | 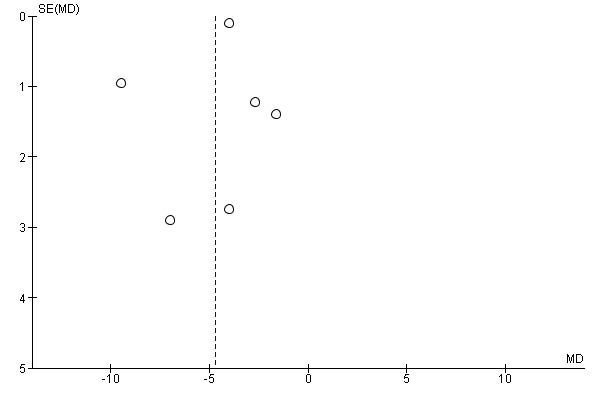 |
| --- | --- |
| (**a**) | (**b**) |
| 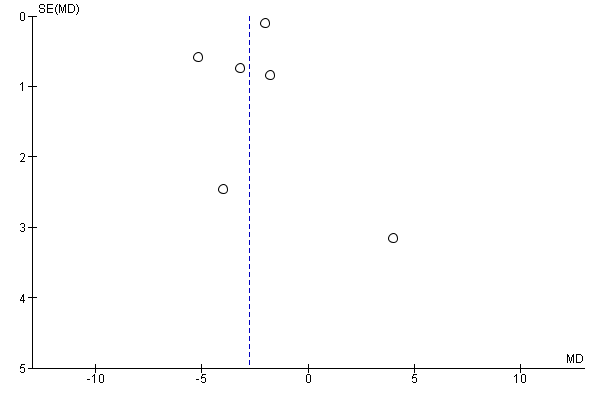 | 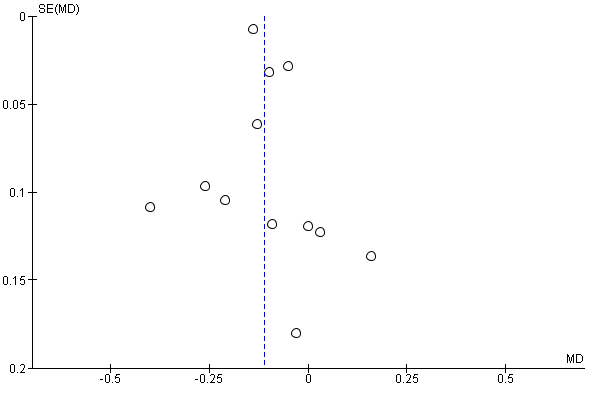 |
| (**c**) | (**d**) |
| 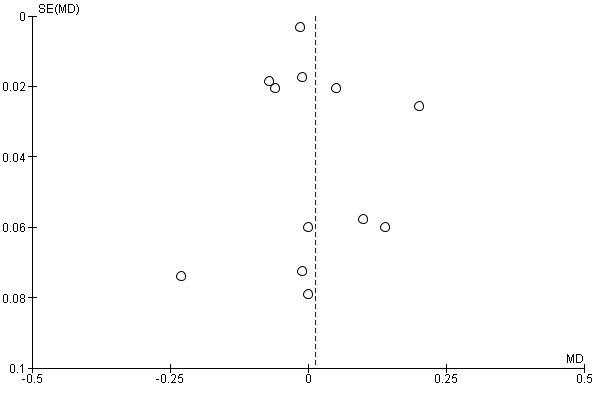 | 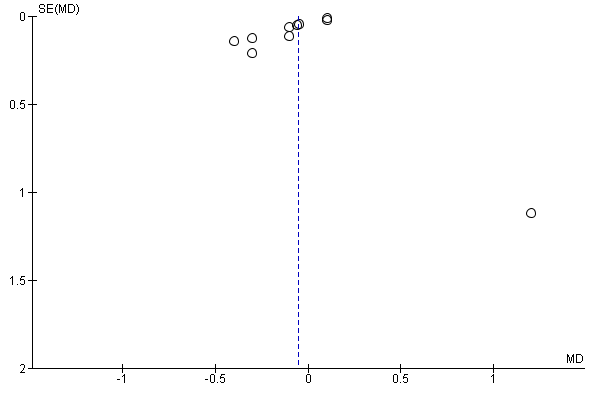 |
| (**e**) | (**f**) |
| 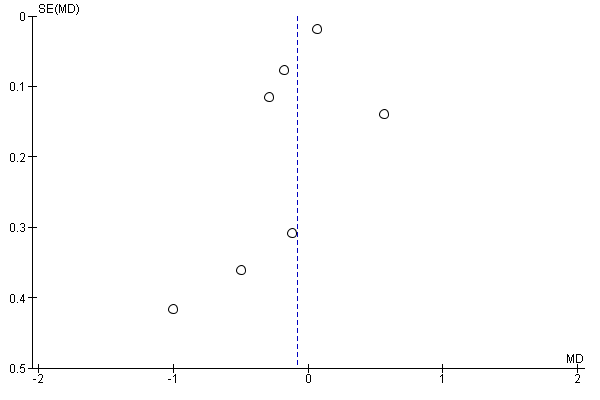 | 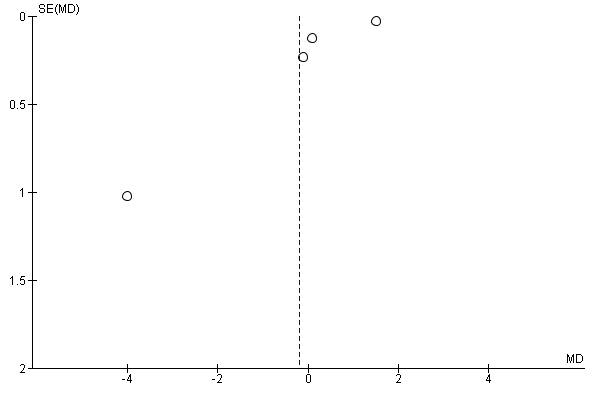 |
| (**g**) | (**h**) |

**Figure S1.** Funnel plot of standard error by standard differences in means of (**a**) Body Mass Index; (**b**) Systolic Blood Pressure; (**c**) Diastolic Blood Pressure; (**d**) Tryglicerides; (**e**) HDL-cholesterol; (**f**) Fasting Glucose; (**g**) HOMA-OR; (**h**) hsCRP.
